# The cognitive profile of middle‐aged and older adults with high vs. low autistic traits

**DOI:** 10.1002/aur.2866

**Published:** 2022-12-01

**Authors:** Gavin R. Stewart, Anne Corbett, Clive Ballard, Byron Creese, Dag Aarsland, Adam Hampshire, Helen Brooker, Rebecca A. Charlton, Francesca Happé

**Affiliations:** ^1^ Institute of Psychiatry, Psychology & Neuroscience King's College London London UK; ^2^ College of Medicine and Health University of Exeter Exeter UK; ^3^ Department of Medicine Imperial College London London UK; ^4^ Department of Psychology Goldsmiths University of London London UK

**Keywords:** aging, ASD, autistic traits, cognition, executive function, information processing, memory, older adults

## Abstract

Cognitive differences in memory, information processing speed (IPS), and executive functions (EF), are common in autistic and high autistic trait populations. Despite memory, IPS and EF being sensitive to age‐related change, little is known about the cognitive profile of older adults with high autistic traits. This study explores cross‐sectional memory, IPS and EF task performance in a large sample of older adults in the online PROTECT cohort (*n* = 22,285, aged 50–80 years), grouped by high vs. low autistic traits. Approximately 1% of PROTECT participants (*n* = 325) endorsed high autistic traits [henceforth Autism Spectrum Trait (AST) group]. Differences between AST and age‐, gender‐, and education‐matched comparison older adults (COA; *n* = 11,744) were explored on memory, IPS and EF tasks and questionnaires administered online. AST had lower performance than COA on tasks measuring memory, working memory, sustained attention, and information processing. No group differences were observed in simple attention or verbal reasoning. A similar pattern of results was observed when controlling for age, and current depression and anxiety symptoms. In addition, AST self‐reported more cognitive decline than COA, but this difference was not significant when controlling for current depression symptoms, or when using informant‐report. These findings suggest that autistic traits are associated with cognitive function in middle‐aged and later life. Older adults with high autistic traits experienced more performance difficulties in a range of memory, IPS and EF tasks compared with the low autistic traits comparison group. Further longitudinal work is needed to examine age‐related change in both older autistic and autistic trait populations.

## Lay Summary

Little is known about the cognitive profile of middle‐aged and older autistic or high autistic trait adults. Using data from the PROTECT cohort and a well‐validated online cognitive assessment platform, the findings from the current study suggest that higher autistic traits are associated with worse cognitive functioning throughout later adulthood. This suggests that older adults with high autistic traits may require additional support as they age.

## INTRODUCTION

Autism represents a highly heritable heterogenous group of lifelong neurodevelopmental conditions, characterized by differences in social communication and restricted‐repetitive behaviors (American Psychiatric Association, 2013). Autism affects ~1% of the population in the UK (Brugha et al., 2016), with an additional 10%–20% endorsing high but subclinical autistic traits (Constantino & Todd, 2003; Wheelwright et al., 2010). There are an estimated 650,000 autistic people in the UK, with approximately 240,000 of these being over 50 years of age (Brugha et al., 2016; Office for National Statistics, 2018). Despite this large number of autistic older adults, autism is under‐researched in this age group and little is known about age‐related cognitive change in the autistic population (Happé & Charlton, 2012; Roestorf et al., 2018; Wise, 2020).

Typical aging is often characterized by a decline in memory (i.e., the ability to retain and recall information), information processing speed (IPS; i.e., the time taken to react to and process information), and in executive function (EF; i.e., an umbrella term for abilities that support goal‐directed behaviors, e.g., planning, set‐shifting, and generating strategies) (Spreng & Turner, 2019). This natural age‐related decline can be observed from approximately 50 years of age, although some domains do decline earlier in adulthood (Hedden & Gabrieli, 2004). Difficulties with some aspects of memory, IPS and EF are also commonly observed in autistic children and young/middle‐aged adults (Desaunay et al., 2020; Habib et al., 2019; Kuo & Eack, 2020). As autistic traits have been associated with an accelerated pace of aging in several physical health biomarkers at age 45 (Mason et al., 2021), and subjective cognitive impairments in older age (Caselli et al., 2018; Stewart et al., 2018; Wallace et al., 2016), autism may confer an additional layer of susceptibility for accelerated cognitive aging later in life.

Two recent reviews of the autistic aging literature have highlighted the dearth of information we have about cognitive aging in autism (Mason et al., 2022; Tse et al., 2021), with Tse et al. (2021) indicating that older autistic adults are likely to have a complex cognitive profile. For visual memory and working memory (the ability to temporarily hold information for further processing), autistic older adults may experience more difficulties than nonautistic individuals (Geurts & Vissers, 2012; Lever et al., 2015; Lever & Geurts, 2016; Ring et al., 2020; Torenvliet et al., 2021). However, for verbal memory, autistic and nonautistic older adults appear to experience similar cognitive profiles (Braden et al., 2017; Lever & Geurts, 2016; Powell et al., 2017; Tse et al., 2019). Conflicting results have also been found for domains of EF, with some studies noting that autistic older adults experience more problems in older age than their nonautistic peers (Braden et al., 2017; Davids et al., 2016; Lever & Geurts, 2016; Powell et al., 2017; Tse et al., 2019), while others show no differences (Geurts et al., 2020; Geurts & Vissers, 2012; Torenvliet et al., 2021; Tse et al., 2019), or suggest (albeit from cross‐sectional data) an improvement with age (Abbott et al., 2018).

From the autistic traits literature, older adults with high autistic traits have been found to experience more subjective and performance‐based difficulties in memory, IPS and most EF domains vs. comparison groups (Caselli et al., 2018; Stewart et al., 2018; Wallace et al., 2016). These performance‐based difficulties in EF domains are also found in older adolescents and younger adults high in autistic traits (Christ et al., 2010; Gökçen et al., 2016). As such, these conflicting findings in memory, IPS, and EFs suggest that autistic (and high autistic trait) older adults are likely to have a complex cognitive profile, with different cognitive strengths and limitations as they age. As such, further work with larger sample sizes is needed to shine a light on the cognitive profile of older adults on the autism spectrum.

Despite this need for further research, there are several challenges and barriers to the study of aging in autism. The first cohort of children diagnosed in the 1960s is only now growing old. Changes to diagnostic criteria over the past 50 years also mean those individuals are not representative of adults diagnosed today (Stuart‐Hamilton et al., 2010). In addition, autism predominately remains the purview of child psychiatrists, thus many autistic older adults remain undiagnosed (Brugha et al., 2016; Office for National Statistics, 2018; Stuart‐Hamilton et al., 2010); referred to as “the lost generation” (Lai & Baron‐Cohen, 2015). Autism is increasingly seen as lying at the end of a dimension of socio‐communicative difficulties, with overlapping genetic influences operating on diagnosed autism and subclinical autistic traits in the general population (Bralten et al., 2018; Whitehouse et al., 2011). Research taking a dimensional, trait‐wise approach to autism is becoming increasingly common; improving statistical power by including large numbers of individuals with high levels of autistic characteristics who nonetheless fall below the diagnostic threshold. This approach may be particularly useful for exploring autism‐related issues in under‐studied and under‐diagnosed groups, such as older adults or in women more broadly. Not only could knowledge of aging in autism improve cognitive theories of the condition, knowing the cognitive strengths and weaknesses of older autistic adults is vital to plan appropriate support (Stuart‐Hamilton et al., 2010; Tse et al., 2021).

The current study investigates the cognitive profile of older adults with high autistic traits in a large sample of adults aged 50 years plus. It is hypothesized that (1) older adults with high autistic traits will demonstrate poorer performance in a range of cognitive assessments (measuring episodic memory, working memory, attention, information processing, and verbal reasoning) than an age‐, education‐, and gender‐matched low autistic traits comparison group. It is also hypothesized that (2) any performance differences in cognitive assessments will persist when controlling for symptoms of current depression and anxiety. In addition, older adults with high autistic traits will (3) self‐report more symptoms of cognitive decline than low autistic trait comparison adults. Finally, (4) cognitive performance will be negatively associated with age in both high and comparison autistic trait groups. Hypotheses were generated based on the existing literature exploring high autistic traits in older age, as well as the existing literature that examines the influence of mental health on cognitive performance.

## METHODS

### Study design

This study uses cross‐sectional baseline data from the PROTECT study (www.protectstudy.org.uk). Inclusion criteria for participation in the PROTECT study are: aged over 50 years, resident in the UK, with good working understanding of English, and able to use a computer with internet access. Participants who have an established diagnosis of dementia prior to registering to PROTECT are excluded. Participants register online and are required to review the study information sheet and to provide consent via an approved online platform. The PROTECT study received ethical approval from the UK London Bridge National Research Ethics Committee (Ref: 13/LO/1578). The aims and hypotheses of this study were publicly preregistered in October 2020 (osf.io/ju9qg).

### Participants

From a total sample of 22,285 participants (Female *n* = 16,387, 73.7%), 325 (1.4%) met our cut‐off criteria for the Autism Spectrum Traits (AST) group; see Measures section below for inclusion criteria. To create a low autism traits comparison older adults (COA) group, from the remaining 21,960 participants, 4537 participants were excluded for endorsing any autistic traits. To match the AST and COA groups on age (mean and range), gender ratio and education history, a further 5728 participants were excluded using random participant selection methods, resulting in 11,695 participants in the COA group. As similar results were obtained when comparing the AST group to all other PROTECT participants (*n* = 21,960), it appears the COA sample selected for no autistic traits was not unrepresentative or unusual. See Table 1 for demographic characteristics.

**TABLE 1:** Demographic characteristics of the COA and AST groups

		Comparison older adults (COA; *n* = 11,695)		AS traits (AST; *n* = 325)		Group difference	Effect size (Cohen's *d/φ*)
Age, years	*M* (*SD*)	61.32	(6.73)	60.88	(6.80)	*F*(1,12,018) = 1.35, *p* = 0.245	0.06 [−0.04–0.17]
	[95% CI]	[61.20–61.45]		[60.14–61.63]			
	Range	50–80		50–80			
Gender	Male: female	3728: 7983		103: 222		*χ* ^2^ = 0.003, *p* = 0.954	0.001
	%	31.8%: 68.2%		31.7%: 68.3%			
Marital status	Married	8324	(71.2%)	195	(60.0%)	*χ* ^2^ = 30.04, *p* < 0.001***	0.050
	Widowed	546	(4.7%)	10	(3.1%)		
	Separated	200	(1.7%)	7	(2.2%)		
	Divorced	1153	(9.9%)	47	(14.5%)		
	Civil Partnership	61	(0.5%)	2	(0.6%)		
	Cohabiting	740	(6.3%)	31	(9.5%)		
	Single	671	(5.7%)	33	(10.2%)		
Education history	School to 16	1676	(14.4%)	56	(17.2%)	*χ* ^2^ = 2.82, *p* = 0.420	0.015
	School to 18	3633	(31.1%)	91	(28.0%)		
	Undergraduate	3950	(33.8%)	111	(34.2%)		
	Postgraduate	2436	(20.8%)	67	(20.6%)		
Current employment status	Employed	5948	(50.9%)	170	(52.3%)	*χ* ^2^ = 8.06, *p* = 0.018*	0.026
	Retired	5399	(46.2%)	137	(42.2%)		
	Unemployed	348	(3.0%)	18	(5.5%)		
Ethnicity	White	11,498	(98.3%)	315	(96.9%)	*χ* ^2^ = 7.91, *p* = 0.095	0.026
	Non‐white	197	(1.7%)	10	(3.1%)		

* *p* < 0.05;

*** *p* < 0.001.

Age, gender ratio, and education history (i.e., matched characteristics) did not differ between the AST and COA groups. Differences between the AST and COA groups were observed in marital status (with AST more often being divorced, cohabiting, or single), and in employment status (with AST more often being employed or unemployed, and COA more often being retired). While not included in the grouping criteria, 24 participants in the AST group and 0 participants in the COA group self‐reported an autism diagnosis.

### Measures

Demographic information was collected using PROTECT's online survey platform, including age, gender, marital status, education history, employment status, and ethnicity.

AST were measured using the PROTECT AST screener questions (Stewart et al., 2020). This screener comprised five yes/no items, asking about childhood (*n* = 2) and current (*n* = 3) socio‐communicative autistic traits. Participants who endorsed both childhood traits plus at least two of the three current traits met criteria for the AST group. Those in the COA group did not endorse any traits. In a separate sample, these screener questions showed good internal consistency (Cronbach's *a* = 0.82), sensitivity (82%) and specificity (94%) for identifying those with an autism diagnosis (Stewart et al., 2020). See Figure S1 for distribution of screener scores in the full PROTECT sample.

Cognitive assessments were conducted using PROTECT and CogTrack's validated online cognitive test package (Corbett et al., 2015; Wesnes et al., 2017). These tasks assess working memory, episodic memory, and EF (including IPS and reasoning) using standardized tasks that have been adapted and validated for online use with older adults. Before each task, participants are visually presented with specific task instructions. The Paired Associates Learning task (Owen et al., 1993), Digit Span task (Huntley et al., 2017), and Self‐Ordered Search task (Owen et al., 1990) were used to measure visual, verbal, and spatial working memory, respectively. A Picture Recognition task (Wesnes et al., 2017) was used to examine visual episodic secondary memory. A Simple Reaction Time task, Choice Reaction Time task, and Digit Vigilance task (Wesnes et al., 2017) were used to assess aspects of attention and IPS, which fall under the EF umbrella. The Verbal Reasoning task (Baddeley, 1968) was used to examine reasoning, which also falls under the EF umbrella. For a comprehensive description of each task and their instructions, please see Supplementary Materials.

Subjective cognitive difficulty and decline was measured using the IQCODE‐SF self‐report and informant‐report versions (Jorm, 1994). All participants completed the self‐report questionnaire, while only a subset of participants had a completed informant‐report questionnaire [COA *n* = 8015 (68.5%); AST *n* = 196 (60.3%)]. The IQCODE‐SF is a 16‐item questionnaire (rated on a 5‐point scale) where participants, or informants, are asked to reflect whether they have improved (=1), stayed the same (=3) or have become worse (=5) at a range of tasks, such as remembering important dates or recent conversations, learning new things, handling money and shopping, and using their intelligence to solve common problems. Scores are averaged (mean scores = 1–5), with a score ≥3.31 indicating that the participant has likely experienced cognitive decline over the past 10 years. The questionnaire and cut‐off have been found to have high reliability for identifying cognitive decline (Jorm, 2004); however, to the authors knowledge, this measure and cut‐off have yet to be validated in an autistic (or high autistic trait) population.

Symptoms of recent depression were measured using the Patient Health Questionnaire (PHQ‐9; Kroenke et al., 2001), and symptoms of recent anxiety were measured with the General Anxiety Disorder questionnaire (GAD‐7; Spitzer et al., 2006). The PHQ‐9 is a nine‐item questionnaire (rated on a 4‐point scale, maximum score = 27) examining low mood over the past 2 weeks. The GAD‐7 is a seven‐item questionnaire (rated on a 4‐point scale, maximum score = 21) examining anxiety symptoms over the past 2 weeks. The PHQ‐9 has been found to have good psychometric properties for assessing depression symptoms in autistic populations, however, the psychometric properties of the GAD‐7 in autistic populations are not known (Cassidy et al., 2018).

### Statistical analyses

All statistical analyses were performed using SPSS (version 25.0; IBM Corp., 2017). Differences between AST and COA in demographic variables and questionnaire responses were analyzed using analysis of variance (ANOVA) and chi‐square (*χ* ^2^) tests. ANOVA was also used to evaluate differences in task scores for the cognitive assessments. Additional analysis of covariance tests were used to account for age and current symptoms of depression and anxiety in the cognitive assessment task scores. Correlation analyses were used to examine associations between age and task scores in each group, with Fisher's *r*‐to‐*z* transformation being used to examine differences in association strength. Additional exploratory analyses were conducted to examine gender differences in task scores. Post hoc regression analyses (not included in the preregistration plans) were conducted to further explore age and gender effects on the cognitive variables in each group. Multiple comparisons were controlled for using the false discovery rate (FDR) method (Benjamini & Hochberg, 1995), with an initial *α*‐value of 0.05 being used. FDR was applied to all *p*‐values produced by analyses reported in the results section, with adjusted *α*‐values being assigned based on the *p*‐value rank. Supplementary analyses (e.g., comparing AST to all other participants, and so forth) were included in a separate FDR ranking table.

## RESULTS

### Cognitive assessments

The AST group had significantly lower mean performance scores than COA on tasks that measured visual, verbal, and spatial working memory (i.e., Paired Associates Learning task, Digit Span task, and Self‐Ordered Search task). The AST group also had lower mean total accuracy ratings on the visual episodic memory task (i.e., Picture Recognition task), and lower mean total accuracy scores, higher mean reaction times, and higher mean false alarm scores in (sustained) attention and information processing tasks (i.e., Choice Reaction Time task, Digit Vigilance task) than the COA group. These differences had small‐to‐moderate effect sizes (Cohen's *d* = 0.17–0.31). However, no differences between AST and COA were observed in mean reaction time for the visual episodic memory task (i.e., Picture Recognition task), in mean simple reaction time (i.e., Simple Reaction Time task), or in mean total score in the reasoning task (i.e., Verbal Reasoning task). See Table 2 for cognitive assessments scores. A similar pattern of results was found when controlling for age, and current symptoms of depression and anxiety. See Table S1 for depression/anxiety adjusted cognitive assessment scores.

**TABLE 2:** Cognitive assessment performance summary scores of the COA and AST groups

Domain	Task	Variable	Comparison older adults (COA; max *n* = 11,695)		AS traits (AST; max *n* = 325)		Group difference	Effect size (Cohen's *d*)
Visual working memory	Paired associates learning (Owen et al., 1993)	Total score (max = 16)	4.61 (0.74)	[4.60–4.62]	4.44 (0.87)	[4.34–4.54]	*F*(1,12,018) = 15.40, *p* < 0.001***	0.22 [0.11–0.33]
Verbal working memory	Digit span (Huntley et al., 2017)	Total score (max = 20)	7.65 (1.47)	[7.63–7.68]	7.20 (1.54)	[7.03–7.37]	*F*(1,12,018) = 28.89, *p* < 0.001***	0.30 [0.19–0.41]
Spatial working memory	Self‐ordered search (Owen et al., 1990)	Total score (max = 40)	7.73 (2.25)	[7.69–7.77]	6.99 (2.74)	[6.69–7.30]	*F*(1,12,018) = 32.34, *p* < 0.001***	0.32 [0.20–0.43]
Cued visual episodic secondary memory retrieval	Picture recognition (Wesnes et al., 2017)	Accuracy rating (%)	91.30 (6.56)	[91.17–91.42]	90.05 (8.01)	[89.10–91.00]	*F*(1,10,533) = 9.59, *p* = 0.002**	0.17 [0.06–0.28]
		Reaction time (ms)	1409 (310)	[1403–1415]	1432 (332)	[1393–1471]	*F*(1,10,533) = 1.44, *p* = 0.231	0.07 [−0.04–0.17]
Attention	Simple reaction time (Wesnes et al., 2017)	Reaction time (ms)	379 (80)	[377–381]	389 (75)	[380–398]	*F*(1,10,589) = 1.05, *p* = 0.305	0.06 [−0.05–0.16]
Attention and information processing	Choice reaction time (Wesnes et al., 2017)	Accuracy score (%)	97.37 (2.35)	[97.33–97.42]	96.78 (2.80)	[96.45–97.12]	*F*(1,10,573) = 16.77, *p* < 0.001***	0.23 [0.12–0.34]
		Reaction time (ms)	525 (67)	[524–526]	540 (71)	[532–549]	*F*(1,10,573) = 14.21, *p* < 0.001***	0.21 [0.10–0.32]
Sustained attention and information processing	Digit vigilance (Wesnes et al., 2017)	Accuracy score (%)	98.00 (3.87)	[97.92–98.07]	97.13 (7.02)	[96.30–97.96]	*F*(1,10,585) = 12.72, *p* < 0.001***	0.20 [0.09–0.31]
		Reaction time (ms)	482 (42)	[481–483]	496 (46)	[491–502]	*F*(1,10,585) = 31.63, *p* < 0.001***	0.31 [0.21–0.43]
		False alarm score	1.86 (1.71)	[1.83–1.89]	2.19 (1.94)	[1.96–2.42]	*F*(1,10,585) = 10.21, *p* < 0.001***	0.18 [0.07–0.29]
Verbal reasoning	Verbal reasoning (Baddeley, 1968)	Total score (max = 64)	33.47 (8.61)	[33.32–33.63]	32.82 (9.97)	[31.72–33.93]	*F*(1,12,018) = 1.73, *p* = 0.188	0.07 [−0.04–0.18]

*Note*: Mean, SD, [95% CI]. Group sizes varied in some tasks due to their point of implementation in PROTECT. Picture recognition, COA *n* = 10,280, AST *n* = 277; Simple reaction time, COA *n* = 10,298, AST *n* = 277; Choice reaction time, COA *n* = 10,292, AST *n* = 277; Digit vigilance, COA *n* = 10,297, AST *n* = 277. All other tasks have max *n* for each group.

** *p* < 0.01;

*** *p* < 0.001.

*Gender differences*: Small gender differences were found in attention and information processing accuracy score (choice reaction time) and sustained attention and information processing accuracy score and false alarm score (digit vigilance), with women having higher accuracy scores and lower false alarm scores than men. No interactions of trait group and gender were found in the ANOVA models. See Table S2 for group performance scores split by gender. In the post hoc regression models, gender was found to be a significant predictor of performance in some cognitive domains for the COA group (with women performing better than men), specifically in accuracy score for the choice reaction time task, in reaction time and false alarm score in the digit vigilance task, and in verbal reasoning score. Gender was not found to be a performance predictor in the AST group for any cognitive task scores. See Table S4 for a breakdown of these post hoc regression analyses by group.

*Age associations*: Using correlation analyses, significant associations were found between age and most cognitive assessment task scores in the COA group; older age was associated with lower performance scores, higher reaction times, and more errors. Fewer significant associations with age were found in the AST group; only paired associates learning total score, picture recognition reaction time, digit vigilance false alarm score, and verbal reasoning were correlated with age (older age, worse performance). Age and task score associations were small‐to‐modest and most did not differ in strength between the AST and COA groups, the exception being a stronger positive age association with reaction time in the choice reaction time and digit vigilance tasks in the COA vs. AST group. See Table S3 for a breakdown of associations by group and group differences. In the post hoc regression models, older age was found to be a predictor of lower scores in all tasks for the COA group and in some tasks for the AST group (e.g., paired associates learning, picture recognition, and verbal reasoning). See Table S4.

*Interactions between gender and age*: Interactions between gender and age: Further post hoc regression analyses were conducted in the AST and COA groups to explore whether there were any interactions between gender and age affecting the cognitive assessment scores in the sample. No significant interactions between gender and age were found in the AST group. In the COA group, significant gender and age interactions were found in choice reaction time accuracy score, and in digit vigilance accuracy score, reaction time, and false alarm score. See Table S4.

### Self‐ and informant‐reports of cognitive difficulty and decline

For the IQCODE‐SF self‐report questionnaire, the AST group reported higher scores (and more above cut‐off scores) for cognitive difficulty and decline than the COA group. However, this group difference was no longer significant when controlling for current symptoms of depression; *F*(1,11,731) = 1.78, *p* = 0.182. For the subset of participants who had a complete IQCODE‐SF informant‐report questionnaire, no group differences were found. See Table 3 for these questionnaire scores.

**TABLE 3:** Questionnaire means, standard deviations, and confidence intervals of the COA and AST groups

Domain		Comparison older adults (COA; *n* = 11,695)		AS traits (AST; *n* = 325)		Group difference	Effect size (Cohen's *d*/*φ*)	Odds ratio
Cognitive decline (self) (max score = 5, cut‐off ≥3.31)	*M* (*SD*)	3.09 (0.23)		3.17 (0.33)		*F*(1,11,925) = 36.61, *p* < 0.001***	0.34 [0.23–0.45]	–
	[95% CI]	[3.08–3.09]		[3.13–3.20]				
	% over cut‐off	1513	(13.0%)	98	(30.5%)	*χ* ^2^ = 81.84, *p* < 0.001***	0.083	2.93 [2.30–3.74]
Cognitive decline (informant)^a^ (max score = 5, cut‐off ≥3.31)	*M* (*SD*)	3.04 (0.23)		3.07 (0.25)		*F*(1,8209) = 2.65, *p* = 0.103	0.12 [−0.02–0.26]	–
	[95% CI]	[3.04–3.05]		[3.03–3.11]				
	% over cut‐off	715	(8.9%)	22	(11.2%)	*χ* ^2^ = 1.24, *p* = 0.265	0.012	1.21 [0.82–2.03]
Depression (max score = 27, cut‐off ≥10)	*M* (*SD*)	2.30	(2.84)	6.07	(5.04)	*F*(1,11,826) = 508.36, *p* < 0.001***	1.26 [1.15–1.37]	–
	[95% CI]	[2.25–2.35]		[5.51–6.63]				
	% over cut‐off	352	(3.1%)	64	(20.4%)	*χ* ^2^ = 270.34, *p* < 0.001***	0.151	8.08 [6.02–10.84]
Anxiety (max score = 21, cut‐off ≥10)	*M* (*SD*)	1.29	(2.30)	4.16	(4.41)	*F*(1,11,901) = 448.78, *p* < 0.001***	1.19 [1.08–1.30]	–
	[95% CI]	[1.24–1.33]		[3.67–4.65]				
	% over cut‐off	155	(1.3%)	34	(10.7%)	*χ* ^2^ = 173.29, *p* < 0.001***	0.121	8.75 [5.93–12.91]

*Note*: Cognitive decline measured using the IQCode self and informant short‐form questionnaire. Depression measured using PHQ‐9; Anxiety measured using GAD‐7.

^a^ 196 AST and 8015 COA participants had a completed IQCode Informant questionnaire. Cognitive Decline (Self) group difference is no longer significant when controlling for current symptoms of depression. The same pattern of results reported above were found in the IQCode (Self) scores when only examining the subset of participants who had completed the informant questionnaire.

*** *p* < 0.001.

Modest agreement was found between self‐ and informant‐reports of cognitive difficulties (AST *r* = 0.24, *p* < 0.001; COA *r* = 0.23, *p* < 0.001), and the strength of these associations was comparable across groups.

*Gender differences*: Some gender differences were found in the informant‐report questionnaire, with men being reported by informant as having more symptoms of cognitive difficulty and decline than women. This gender difference was not found in the self‐report questionnaire. In addition, no interactions between trait group and gender were found for either self‐ or informant‐report questionnaires. See Table S5 for group questionnaire scores split by gender.

*Age associations*: Age and cognitive difficulty and decline associations were small and did not differ in strength between the AST and COA groups for either self‐ or informant‐report questionnaires (*r* self = 0.01–0.04; *r* informant = 0.09–0.16).

## DISCUSSION

The current study documents performance‐based similarities and differences in the cognitive profile of over 300 adults aged 50–80 years with high autistic traits, compared with a large age‐, gender‐, and education‐matched low autistic trait group. Older adults with high autistic traits were found to have lower performance scores than the comparisons group on cognitive assessments measuring working memory (Paired Associates Learning task, Digit Span task, Self‐Ordered Search task), cued episodic secondary memory retrieval (Picture Recognition task), and (sustained) attention and information processing (Choice Reaction Time task, Digit Vigilance task). However, no differences were observed between the high trait vs. low trait older adults in cued episodic secondary memory retrieval reaction time (Picture Recognition task), attention speed (Simple Reaction Time task), or reasoning scores (Verbal Reasoning task). A similar pattern of results was found when accounting for age and current symptoms of depression and anxiety. The findings from the current study suggest that the complex profile of memory, IPS and EF similarities and differences found in other studies that examine autistic older adults also extends to those who do not have an autism diagnosis but nonetheless endorse high autistic traits.

The first key finding in the current study is that older adults with high autistic traits had lower performance scores in a range of visual (Paired Associates Learning task), verbal (Digit Span task), and spatial (Self‐Ordered Search task) working memory tasks compared with those with low autistic traits, with the pattern of results persisting when controlling for age and mental health problems. The findings in our current study are consistent with the lower working memory performance scores reported in previous samples of autistic adults across different age‐ranges by Geurts and Vissers (2012; *m* = 64 years, 21% female) and Lever et al. (2015; *m* = 48 years, range = 20–79, 29% female), and in older adults with high autistic traits by Stewart et al. (2018; *m* = 73 years, range = 60–91, 55% female). These findings from studies that include middle‐aged to older autistic and high autistic trait adults suggest that working memory problems documented throughout adulthood are likely to persist into older age. While the current study found that both high and low autistic trait groups had comparable negative associations between age and performance scores for working memory (as demonstrated in correlation and post hoc regression analyses), as the high autistic traits group had lower scores than the low trait comparison group, these cross‐sectional findings could suggest that working memory may be implicated in autistic cognitive aging. Furthermore, as these working memory tasks include aspects of memory (retention and recall) as well as executive functioning (manipulation of information), it is important to consider these findings alongside the other memory and EF findings and relevant literature outlined below.

The second key finding in the current study is that older adults with high autistic traits had lower performance scores in visual episodic memory (Picture Recognition task) compared with those with low autistic traits, with the pattern of results persisting when controlling for age and mental health problems. Furthermore, age was not found to be a predictor of task accuracy in either group. Our pattern of results regarding visual memory are not as clearly consistent with the existing literature. In the current study, we found that older adults with high autistic traits had lower accuracy scores than comparisons with low autistic traits on a task that measures cued visual episodic memory retrieval (Picture Recognition task); however, no differences were found in reaction time for this task. These findings are consistent with the lower performance scores reported by Tse et al. (2019), who had a similarly aged sample of autistic adults (*m* = 61 years, age range = 50–72, 21% female), suggesting that visual memory could be implicated in autistic cognitive aging. However, Lever et al. (2015) reported that autistic adults (*m* = 48 years, range = 20–79, 29% female), including a subsample aged 50–79 years, had higher performance scores in this domain than nonautistic comparisons, suggesting that visual memory could be a strength in autistic cognitive aging. These inconsistencies could be due to differences in the sample demographics, particularly age, between studies. While our cross‐sectional data suggest comparable effects of age in the high and low autistic trait groups (as demonstrated in correlation and post hoc regression analyses), further research (including longitudinal studies) into visual memory processes is required to explore the influence of aging on this domain in relation to autism/autistic traits.

The third key finding in the current study is that older adults with high autistic traits had lower performance scores on more complex attention and IPS tasks compared with those with low autistic traits, with the pattern of results persisting when controlling for age and mental health problems. These lower scores and accuracy ratings are found in tasks that measure sustained attention and IPS (Choice Reaction Time and Digit Vigilance tasks), but not in tasks that measure simple attention/reaction time (Simple Reaction Time task). Increased reaction times were found to be associated with age in both groups in correlation analyses, but the strength of this association was stronger in COA. In addition, age was only found to be a predictor of reaction time in the post hoc regression models for COA but not AST, suggesting a possible different pattern of age‐related change. For sustained attention, the findings in our current study are consistent with the findings of Geurts and Vissers (2012) in their sample of autistic adults. In addition, our findings of slower IPS and accuracy are consistent with the findings of Lever and Geurts (2016; *m* = 48 years, range = 20–79, 29% female), Davids et al. (2016; *m* = 58 years, range = 50–84, 16% female), and Powell et al. (2017; *m* = 49 years, range = 30–67, 17% female) in their samples of autistic adults, and in the high autistic traits sample in Stewart et al. (2018; *m* = 73 years, range = 60–91, 55% female). Simple attention reaction time was not found to differ from the comparison group in the current study, which is consistent with the findings of Lever and Geurts (2016). Overall, findings suggest that the sustained attention (but not simple attention) and information processing problems documented throughout adulthood are likely to persist into older age for those on the autism spectrum, but reaction times may be preserved.

The fourth key finding in the current study is that older adults with high autistic traits had comparable scores in reasoning (Verbal Reasoning task) compared with those with low autistic traits, with the pattern of results persisting when controlling for age and mental health problems. While this suggests that the high and low autistic trait groups in the current study are approximately matched on IQ, this finding is inconsistent with the previous literature examining verbal reasoning performance. Bertrams and Schlegel (2020; *m* = 38 years, range = 21–72, 46% female), who also used Baddeley's Verbal Reasoning task, found a modest negative association between verbal reasoning scores and autistic traits. Several other studies examining EF more broadly (which is closely linked to verbal reasoning) have found that older adults with high autistic traits (e.g., Stewart et al., 2018; *m* = 73 years, range = 60–91, 55% female), and middle‐aged and older autistic adults (e.g., Lever & Geurts, 2016; *m* = 48 years, range = 20–79, 29% female; Davids et al., 2016; *m* = 58 years, range = 50–84, 16% female; Powell et al., 2017; *m* = 49 years, range = 30–67, 17% female), experience more EF performance problems than nonautistic or low autistic trait comparison groups. In the current study the high and low autistic trait groups showed similar patterns of (cross‐sectional) negative association between verbal reasoning and age (as demonstrated in correlation and post hoc regression analyses). Further research is required using verbal reasoning and fluid intelligence measures (chosen carefully, given evidence of large differences by measure in autism; Barbeau et al., 2013) to understand the influence of autistic traits on verbal reasoning, and EF more broadly, in older age.

The fifth and final key finding in the current study was that older adults with high autistic traits self‐reported more subjective cognitive difficulties when compared with those with low autistic traits. Informant‐reports, available for a subset of the sample, did not follow this pattern, and showed no group difference. This finding of increased self‐reported cognitive difficulties is consistent with Davids et al. (2016; *m* = 58 years, range = 50–84, 16% female), Lever and Geurts (2016; *m* = 48 years, range = 20–79, 29% female), and Geurts et al. (2020; *m* = 66 years, range = 60–85, 0% female) autistic older adult studies, and in the high autistic trait samples in Wallace et al. (2016; *m* = 74 years, range = 61–88, 45% female), Stewart et al. (2018; *m* = 73 years, range = 60–91, 55% female), and Caselli et al. (2018; *m* = 68 years, range = 40–80, 51% female). However, the different pattern in self‐ and informant‐reported cognitive difficulties suggests self‐appraisal of current cognitive functioning may be overly negative. As the difference in self‐reported cognitive functioning was no longer significant when accounting for symptoms of depression, poor mental health may be driving this negative self‐appraisal. In the current study, weak negative associations were found between age and self‐ and informant‐reported cognitive difficulties and task performance in both high and low autistic trait groups. However, these weak age associations could also be due to the restricted age‐range in the current study, with half of the participants in both high and low autistic trait groups being under 60 years of age.

When thinking about the clinical implications of these findings, several points are worth consideration. First, differences between the AST and COA groups were found on some (e.g., working memory, and so forth) but not all (e.g., reaction time, and so forth) cognitive domains. In addition, the AST group self‐reported more general cognitive difficulties in their day‐to‐day lives. These findings indicate that middle‐aged and older people with high autistic traits are likely to have both an uneven cognitive profile and more general functional difficulties, which could have broader implications across their lifespans. The current sample is of retirement age and further demographic information is not available to probe the effect of an uneven cognitive profile on employment success. However, autistic people have been found to generally have worse outcomes (e.g., lower rates of employment, independent living) than their nonautistic peers (Mason et al., 2021). It could be hypothesized that this uneven cognitive profile and general functional difficulties play a role in these poorer outcomes, which warrants further investigation (that utilize longitudinal designs) across the lifespan to examine how cognitive profile/change and other detrimental experiences widely reported in autistic/high autistic trait populations (e.g., poor mental health) influence life outcomes.

Second, it is important to note the effect size of the statistically significant differences reported in this study. The performance score differences between the groups have small‐to‐moderate effect sizes; while these differences are statistically significant, it is questionable whether they would have a significant clinical impact at a group level. However, future longitudinal research tracking individual cognitive trajectories, and experiences of everyday functioning, across time would provide vital information about cognitive aging on the autism spectrum, which in turn could provide useful guidance to ensure support needs are met.

Third, it is important to note that this sample is comprised of middle‐aged and older adults with high autistic traits, not those with an autism diagnosis. Many middle‐aged and older adults who would meet diagnostic criteria for autism remain undiagnosed due to changes in the diagnostic criteria for autism over their lifetime; sometimes referred to as the “lost generation” (Lai & Baron‐Cohen, 2015). While the authors believe that information about aging with high autistic traits can be informative about autistic aging, studies of this nature do not replace those with participants with clinically verified autism diagnoses.

When contextualizing the findings of this study, it is important to consider strengths and limitations of the design and methodologies used. A strength of PROTECT is its use of an online platform, allowing large scale recruitment from a wide geographical spread across the UK. In addition, the use of the well‐validated online platform for cognitive assessments allows for a large amount of rich, objective data to be collected about a broad range of cognitive domains without the need for in‐person assessments. However, this also poses a limitation for the current study, as older adults who do not feel comfortable using a computer or who do not have access to the internet would not be able to participate. Older adults who engage in medical research are typically those who are more physically and mentally able, which may lead to sampling biases, survivor effects, and poor generalizability of findings (Golomb et al., 2012). In PROTECT, as in most volunteer samples, females are over‐represented (~68% of the sample). Although our groups were matched on gender‐ratio, our findings may not generalize to all gender identities. In addition, the PROTECT sample is predominately white (~98%), and does not reflect the current breakdown of ethnicities found in the UK (~85% white; Office for National Statistics, 2021). Furthermore, given the cross‐sectional nature of this study, it is not possible to determine whether the cognitive differences reported in this study are longstanding or the effect of age‐related change; further research that utilizes longitudinal designs is needed to examine the trajectories of age‐related change. While attempts were made to examine possible interactions between age and gender in separated autistic trait groups, the authors' ability to conduct more complex analyses were limited due to the smaller size of the AST group. Future studies should consider sample size and power requirements when designing their studies, and consider conducting analyses (e.g., three‐way interaction regressions) that can better examine the relationship between age, gender, and autistic traits. Finally, the criteria used to identify the AST group was a short, bespoke (albeit validated) set of questions rather than a standardized measure. While the screener was found to correlate and have good cut‐off overlap with widely used existing measures of autistic traits (the AQ‐10, RAADS‐14) in a separate validation study (Stewart et al., 2020), the questions included in this screener focus solely on socio‐communicative difficulties, without items probing restrictive/repetitive behaviors or sensory problems. It is possible that participants may have scored highly on this screener for reasons other than autism‐related traits; for this reason, we controlled for depression and anxiety in our analyses. Whilst all these factors may limit the overall generalizability of the findings, the results still provide important, albeit preliminary, new information about the similarities and differences in the cognitive profile of a large population of older adults with and without high autistic traits.

In conclusion, our study exploring the cognitive profile of middle‐aged and older adults suggests both similarities and differences in those with high vs. low autistic traits. This pattern is complex, and it is likely that people with high autistic traits will experience different trajectories of cognitive aging compared with those found in typical aging. The current study is cross‐sectional, and future research using longitudinal designs is needed to study the trajectories of age‐related change to cognitive function in autistic and high trait groups. Furthermore, as older age often represents a period when support needs change, these findings highlight that older adults on the autism spectrum—including those with high traits who may not have a diagnosis—may require additional support from family and autism‐aware services as they enter later life.

## AUTHOR CONTRIBUTIONS

Anne Corbett, Clive Ballard, Byron Creese, Dag Aarsland, Adam Hampshire, Helen Brooker conceived the PROTECT study and have overseen data collection and management. Gavin R. Stewart, Rebecca A. Charlton, and Francesca Happé conceived the presented study. Gavin R. Stewart conducted statistical analyses and wrote the manuscript under the supervision of Rebecca A. Charlton and Francesca Happé, with consultation from other authors. Rebecca A. Charlton and Francesca Happé have verified the underlying data. All authors reviewed the final manuscript.

## Supporting information


**APPENDIX S1:** Supporting Information

## Data Availability

Research data are not shared.
